# Application of Matrix-Assisted Laser Desorption/Ionization Mass Spectrometry Imaging for Evaluating the Quality of Fish Fillets

**DOI:** 10.3390/foods9040402

**Published:** 2020-04-01

**Authors:** Mizuki Morisasa, Keisuke Kimura, Motoki Sumida, Saya Fukumoto, Tadashi Tamura, Riko Takeuchi, Tsukasa Mori, Naoko Goto-Inoue

**Affiliations:** 1 Department of Marine Science and Resources, College of Bioresource Sciences, Nihon University, 1866 Kameino, Fujisawa, Kanagawa 252-0880, Japan; xx1mizu9xx@gmail.com (M.M.); mucunguiyou412@gmail.com (K.K.); mori.tsukasa@nihon-u.ac.jp (T.M.); 2 Central Research Institute, Maruha Nichiro Corporation, 16-2, Wadai, Tsukuba, Ibaraki 300-4295, Japan; s-sumida@maruha-nichiro.co.jp (M.S.); s-fukumoto@maruha-nichiro.co.jp (S.F.); ta-tamura@maruha-nichiro.co.jp (T.T.); ri-takeuchi@maruha-nichiro.co.jp (R.T.)

**Keywords:** matrix-assisted laser desorption/ionization, mass spectrometry imaging, formaldehyde, protease, peptide

## Abstract

Consumption of fish is rapidly increasing worldwide. It is important to evaluate fish fillet quality because fish undergoes physical and chemical changes during frozen storage. Fish fillets exhibit formaldehyde (FA) accumulation from the decomposition of trimethylamine N-oxide. FA is a powerful protein denaturant; thus, it is important to avoid FA buildup during fish processing to preserve fish quality, especially texture. To determine where FA accumulates, in order to maintain the quality of fish fillets, we performed matrix-assisted laser desorption/ionization mass spectrometry imaging, aiming to identify muscle-derived peptides, which reflect conditions such as denaturation and/or aggregation. We used frozen sections from which lipophilic molecules were washed out and detected various peptide peaks. Furthermore, we tried to identify indices to represent fish fillet softening by protease treatment. We could detect characteristic peaks owing to FA and protease treatment; the findings were consistent with the results of texture profiles showing fish fillet’s real solidity. These molecules might thus serve as effective markers to evaluate fish fillet quality.

## 1. Introduction

**Figure 1:**
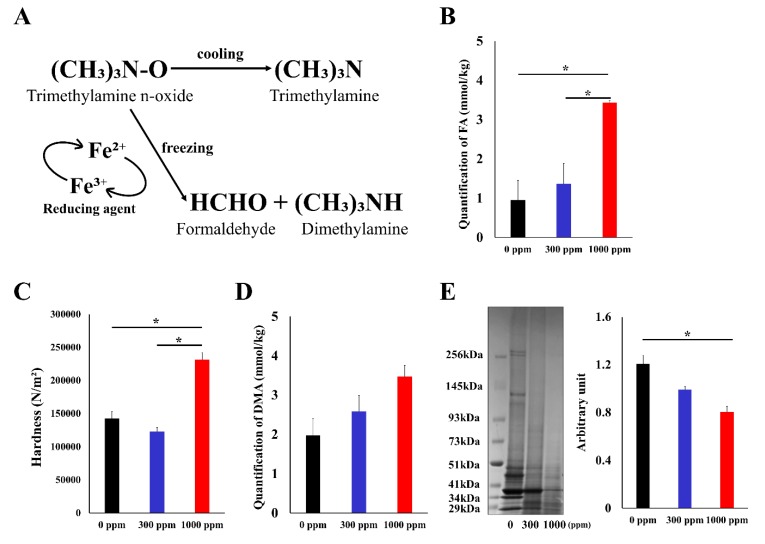
(**A**) Scheme of the production of dimethylamine (DMA) and formaldehyde (FA) from trimethylamine N-oxide (TMAO) in fish muscles. (**B**) Quantification of FA in three different fish fillet samples. (**C**) Measurement of hardness (N/m^2^) using the texture analyzer. (**D**) Endogenous FA from the amount of DMA. The amount of DMA did not change among these samples. (**E**) SDS-PAGE analyses and quantitative measurements of extracted proteins. *: Differences with *p* < 0.05 were considered statistically significant.

The global population is over 7 billion people, and providing a sustainable food supply chain is an urgent issue. Food softening treatments, such as protease treatment, have recently been established [1]. Foods treated by these methods can be easily swallowed and are commonly served to elderly persons to maintain their quality of life [2]. In Japan, the market has expanded by about 1.5-fold over the last five years and is expected to expand further in the future [3]. Consumption of fish has been rapidly increasing worldwide, and fish filleting is often performed in the abovementioned food softening method. With this treatment, a protease is introduced into a food within a short period by immersing and thawing in a protease solution and reducing pressure [4]. After an enzymatic reaction, the food is softened while maintaining its original structure. This method has been applied to prepare foods that can be easily swallowed but maintain their natural features, nutrients, and flavor components, even after cooking [5]. However, fish is one of the most perishable and vulnerable marine products. It is reported that cold storage for 15 days worsens the quality of fish fillets due to the hydrolysis of myofibrillar protein [6,7]. In particular, it is known that cod (Gadiformes) undergoes rapid postmortem changes and has low cryo-preservability [8]. For this reason, the hardening of fish meat may reduce the quality of fish fillets. One of these causes is the decomposition of trimethylamine N-oxide (TMAO) [9]. TMAO is an osmotic-pressure-controlling compound found in fish meat or blood and is produced in an equivalent amount to dimethylamine (DMA), which causes a foul fish smell [10], and formaldehyde (FA) in fish muscles (Figure 1A) [11,12]. Particularly, FA is a powerful protein denaturant that causes fish meat to harden; consequently, it is important to avoid FA accumulation during fish processing to preserve fish quality [13]. To measure the buildup of FA, classical analytical methods such as quantification of TMAO and FA have been used; however, these methods are time-consuming and are not highly sensitive. Previously, Shumilina et al. described the quality of salmon fillets using high resolution nuclear magnetic resonance spectroscopy [14]. They could detect many metabolites, but the localization of these metabolites were lost during the process of extracting the tissue. FA accumulation occurs locally, and evaluating the localization of FA is difficult [15,16]. We realized that it is very important to determine where FA accumulates to maintain the quality of fish fillets [17], and another methodology is needed for this demand.

We previously used mass spectrometry (MS) imaging of fish fillets to detect dipeptides and demonstrate their localization [18]. In the present study, we aimed to evaluate fish quality using MS imaging to analyze the localization of peptides digested from muscle proteins. We used two models; one was to measure the hardness of fish fillets affected by FA that were immersed in an FA solution and to compare the peptide detection among different FA concentrations. The other model was constructed to detect molecular changes occurring due to the treatment of protease with and without FA. We then attempted to identify the biochemical indices that could be used to evaluate the quality of fish fillets.

## 2. Materials and Methods

### 2.1. Samples

*Macruronus novaezelandiae* were imported from Kingfisher Holdings (Maruha Nichiro Co. Ltd., Bangkok, Thailand) and stored at −30 °C.

### 2.2. FA Processing

FA (064-00406, FUJIFILM Wako Pure Chemical Corp., Osaka, Japan) solutions were prepared as follows. FA 1000 ppm were prepared with 1.3 mL of 37% formaldehyde adjusted to 500 mL with water. FA 300 ppm was adjusted by diluting the FA 1000 ppm solution with water. After tenderization, samples were immersed for 30 min in 0 ppm (distilled water), 300 ppm, or 1000 ppm FA solutions and stored at −10 °C for 1 week in a vacuum pack (C200 MULTIVAC, Memmingen, Germany).

### 2.3. Protease Treatment

Fish fillets were soaked in a 0.3% enzyme solution (Multifect PR6L A01440G191, Genencor, Palo Alto, CA, USA) under reduced pressure; the pressure was reduced to 10 kPa for 5 min and then returned to atmospheric pressure, and this process was repeated four times. For the enzymatic reaction, fish fillets were stored overnight at 4 °C [4,19]. To obtain the autolysis-generated peptide fraction, enzymatic reaction was performed at 37 °C for 1 h.

### 2.4. Texture Profile Analysis (TPA)

Fish fillets were thawed at room temperature (25 °C), and then heated in a steam convection oven (MIC-5TB3, Hoshizki Corp., Aichi, Japan) at 160 °C for 14 min and then cooled to room temperature. The texture analyzer (EX-SX 500N, Shimadzu, Kyoto, Japan) was set in compression mode. After the probe was allowed to touch the sample surface, the test began with the probe (diameter of 20 mm) moving at 10 mm/s and compressing the tissue to 30% of its original height at room temperature [20].

### 2.5. Quantification of FA in Fish Fillets

The samples (2.0 g) were stirred with a disperser for approximately 30 s after adding 10 mL of 20% trichloroacetic acid (TCA). The supernatants were filtered with a syringe filter (0.2 μm) after centrifugation for 30 min at 5000 × *g*. The samples were then diluted with water and the FA standards (064-00406, FUJIFILM Wako Pure Chemical Corp., Osaka, Japan) at different concentrations (0.020, 0.20, 0.40, and 1.0 ppm) were then mixed with 5 N potassium hydroxide solution (2.0 mL) and 4-amino-3-hydrazino-5-mercapto-1,2,4-triazole solution (2.0 mL) and maintained at room temperature for 15 min. After adding potassium periodate solution (2.0 mL), they were mixed until the foaming ceased. FA was quantified by measuring the absorbance at 550 nm using a spectrophotometer (U-5100, Hitachi High-Technologies Corp., Tokyo, Japan) [21].

### 2.6. Quantification of DMA in Fish Fillets

The samples (2.0 g) were stirred with a disperser for approximately 30 s after adding 20 mL of 5% trichloroacetic acid (TCA). The supernatants were filtered with a syringe filter (0.2 μm) after centrifugation for 5 min at 4000 × g. The samples (1.0 mL) were diluted with 1.0 mL of water and 1.0 mL of 2% TCA, and the dimethylamine hydrochloride standard solutions (1.0 mL) (20230-1210, Junsei Chemical Co. Ltd., Tokyo, Japan.) at various concentrations (0, 0.781, 1.563, 3.125, and 6.250 ppm) were diluted with 1.0 mL of water and 1 mL of 5% TCA. Both solutions were stirred with a super mixer for 1 min after adding chloroform–carbon-disulfide (a rate of 5%) mixture and alkaline reagent (0.1 mL, 1:1 mixed solution of sodium hydroxide and ammonium hydroxide) and stirred again with a super mixer for 1 min after adding copper reagent (0.5 mL). After manually mixing 30% acetate (0.5 mL), the lower fraction was mixed with sodium sulfate, and the absorbance was measured at 435 nm using a spectrophotometer (U-3900H, Hitachi High-Technologies Corp., Tokyo, Japan) and calculated by colorimetric quantitation [22].

### 2.7. Sodium Dodecyl Sulfate Polyacrylamide Gel Electrophoresis (SDS-PAGE)

The SDS-PAGE analysis was performed as described previously [23]. Briefly, frozen tissues were homogenized in RIPA buffer (Pierce, Thermo Scientific, San Jose, CA, USA) and boiled for 2 min at 98 °C. Homogenates were then centrifuged at 16,000 × g for 40 min at 4 °C, and the total protein concentration of the supernatant was measured using a BCA Protein Assay kit (Pierce, Thermo Scientific). Samples were diluted in Laemmli sample buffer and boiled for 10 min at 98 °C. Protein samples were then separated by SDS-PAGE (5%‒10% polyacrylamide gradient gels), and Coomassie Brilliant Blue staining was used to visualize the proteins. Molecular identification was performed by comparison with molecular weight standards (Multi-color, Nacalai Tesque, Japan).

### 2.8. Matrix-Assisted Laser Desorption/Ionization (MALDI) MS Imaging

Tissue sections (18 µm) were serially cut using a cryostat (CM1950, Leica Microsystems, Wetzlar, Germany). The sections were then mounted onto indium tin oxide coated glass slides (Bruker Daltonics, Germany) for MS imaging. The samples were prepared according to a previously published method [24] after being dipped into methanol to wash away any lipophilic molecules. Appropriate matrix solutions, including 50 mg/mL 2,5-dihydroxybenzoic acid in methanol/water (8:2, v/v), were used. The matrix solution (2.0 mL) was sprayed uniformly over the frozen sections using an airbrush with a 0.2 mm nozzle (Procon Boy FWA Platinum, Mr. Hobby, Tokyo, Japan). MS imaging analyses were performed using TOF/TOF 5800 (AB SCIEX, Framingham, MA, USA) in positive-ion mode for masses in the range of *m*/*z* 500‒2000. The ion images were constructed using the Datacube Explorer Software [25].

### 2.9. Statistical Analysis

All data are expressed as the mean ± standard error of the mean (SEM). Statistical analyses were performed using a Student’s t-test and Bonferroni/Dunn test using StatView 5.0 (SAS Institute, Tokyo, Japan). *: Differences with *p* < 0.05 were considered statistically significant.

## 3. Results and Discussion

To create a sample simulating FA accumulation in fish fillets, we prepared three different fish fillet samples that had been soaked in 0, 300, and 1000 ppm FA. Although there were no significant visual changes in the samples, quantification of FA revealed a significant accumulation of FA in the 1000 ppm sample (Figure 1B). Using a texture analyzer to measure hardness (N/m^2^), we found that the sample that had been soaked with 1000 ppm FA was significantly harder than the one that had been soaked with 0 ppm FA (Figure 1C). FA might be produced from endogenous TMAO, and the amount might differ among samples [8]. To evaluate endogenous FA, we measured the amount of DMA, which is produced in an equal amount from TMAO [26,27]. As a result, there was no change in DMA, suggesting that FA concentration reflects the difference in artificial FA rather than endogenous FA produced from TMAO (Figure 1D). FA accumulation in fish fillets causes fixation of fish proteins and reduces the levels of soluble proteins [7]. To assess this, we extracted soluble proteins from the different FA-containing samples and compared the protein patterns by SDS-PAGE. As shown in Figure 1E, the higher the FA concentration, the lower the quantity of extracted proteins. Particularly, high-molecular-weight proteins such as myosin heavy chains (higher than 250 kDa) and actinin (around 103 kDa) were reduced in the 1000 ppm samples, predominantly.

**Figure 2:**
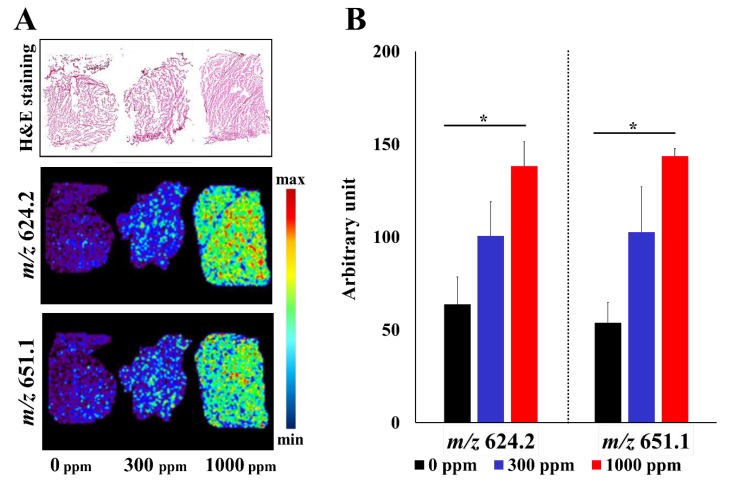
(**A**) MS imaging of three different fish fillet samples (0, 300, and 1000 ppm FA concentrations). The ion images at *m*/*z* 624.2 and *m*/*z* 651.1 are shown. (**B**) The results of semiquantitative analyses of ion intensities are also shown (*n* = 3). *: Differences with *p* < 0.05 were considered statistically significant.

We utilized MS imaging to explore new biochemical indicators of fish “hardness” due to protein aggregation. In this method, to focus on changes in proteins and peptides, we washed the sections with methanol before measurement. After washing with methanol, lipophilic molecules, such as lipids, were washed out. We analyzed peaks at *m*/*z* 500–2000, where peptides are expected to be observed. Consequently, we detected peaks at *m*/*z* 624.2 and *m*/*z* 651.1, the intensity of which increased according to the concentration of FA. The intensities were observed uniformly throughout the fillets (Figure 2), suggesting that these molecular signals could be used as markers of FA accumulation.

**Figure 3:**
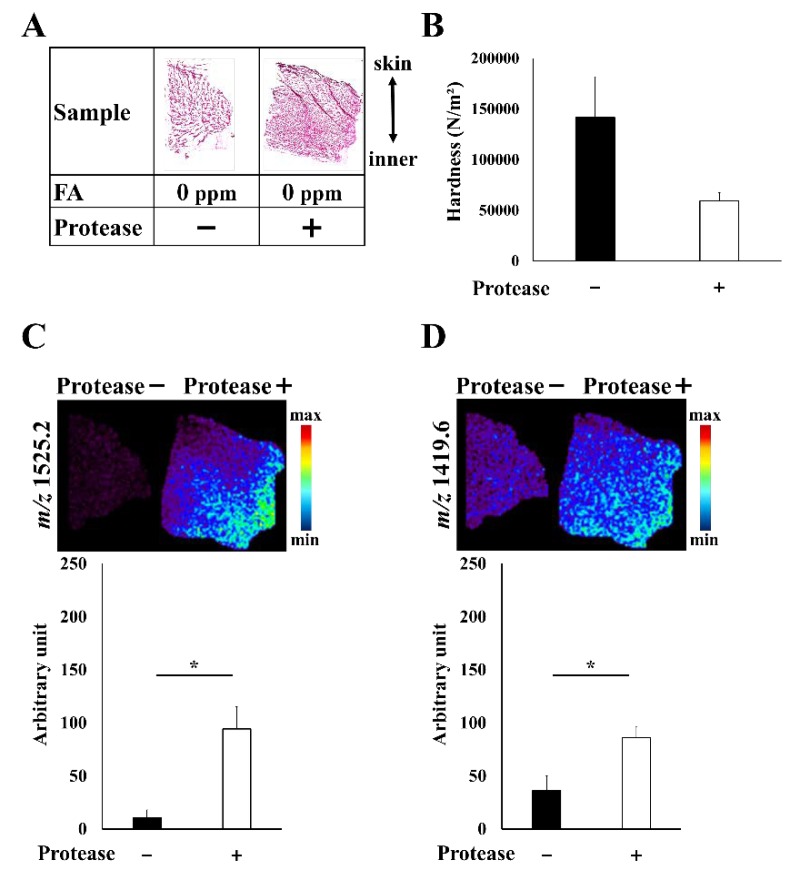
(**A**) Sample conditions—samples treated with and without protease. (**B**) The hardness value was reduced after treatment with protease. (**C** and **D**) MS imaging results in *m*/*z* 1525.2 and *m*/*z* 1419.6 between two different fish fillet samples. *: Differences with *p* < 0.05 were considered statistically significant.

As shown in Figure 3, a fish fillet sample that exhibited a hardness value of approximately 150,000 N/m^2^ was reduced to approximately 50,000 N/m^2^ after treatment with a protease. This was found to be within the standard value for food that is easy to swallow under the guidelines of Japan Universal Design Foods (Figure 3A,B) [2]. After protease treatment, numerous new peaks were detected (Supplemental Figure S1). We focused on *m*/*z* 1525.2 because of its high intensity and reproducibility (Figure 3C). Simultaneously, we monitored *m*/*z* 1419.6, which was a peptide peak derived from the protease itself due to protease autolysis (Supplemental Figure S2, Figure 3D). The peak at *m*/*z* 1525.2 was significantly higher in samples treated with protease. Interestingly, the signal was higher in the inner part of the fish fillet, which demonstrated the degree of penetration of the protease. Therefore, the localization of this peptide reveals the quantity and location of the protease that induced softening.

**Figure 4:**
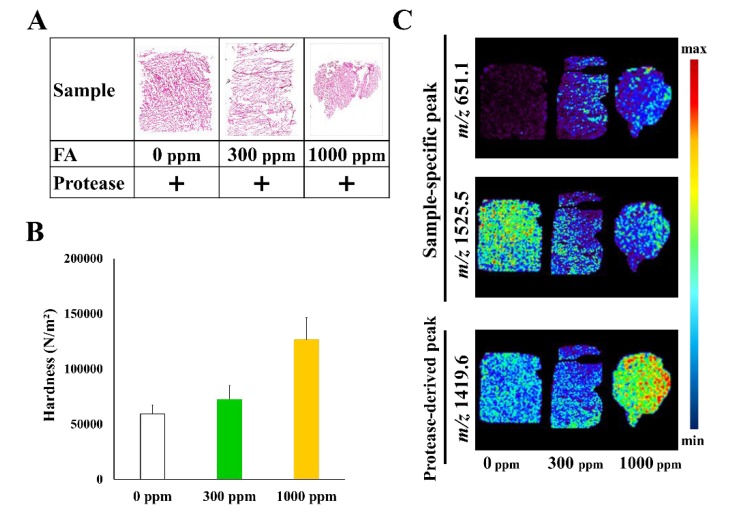
(**A**) Samples treated with protease at three different FA conditions: 0, 300, and 1000 ppm. (**B**) Hardness value. (**C**) Molecular distribution of the ions at *m*/*z* 651.1, 1525.2, and 1419.6.

Finally, we also examined the effect of protease treatment in the presence of FA. As shown in Figure 4, in the 1000 ppm FA sample, the texture profile value was considerably higher than that in the 0 and 300 ppm samples, even with the same protease treatment. Therefore, we attempted to identify molecules that correlated with hardness value by MS imaging. A molecular ion at *m*/*z* 651.1, which was found to be an FA-dependent ion, was detected regardless of the presence of the protease; however, the signals were significantly lower than those seen without protease (data not shown). Contrastingly, the ion at *m*/*z* 1525.2 (which, as described above, is an index of food softening) was found to have a tendency to be inversely correlated with the hardness value. This result suggested that although the protease was active in all fish samples, the protease activities in the presence of FA were lower than those in samples without FA. We hypothesized that the presence of peptides at *m*/*z* 1525.2 and their intensities might be an index for food softening. A protease-derived peptide at *m*/*z* 1419.6 was detected in all the samples, and its intensity was the strongest in the 1000 ppm FA samples. This suggested that muscle-derived proteins were aggregated by FA and could easily be penetrated by protease (Figure 4).

We have shown that some peaks were FA dependent. Interestingly, the results of these peaks and texture profile analyses were consistent. Therefore, the use of MS imaging enables an analysis of the localization of molecules as well as the detection of muscle-derived peptides. In real samples, the production of FA occurs locally, depending on the concentration of TMAO. MS imaging, which is capable of assessing molecular localization, might be a powerful tool for validating the degradation of TMAO. Our data indicate that peptides derived from fish muscles could serve as biochemical molecular markers that could be used to monitor fish fillet conditions.

## 4. Conclusions

In this study, we used MS imaging to identify a new biological index to assess fish fillet quality. We successfully identified two ions at *m*/*z* 651.1 and *m*/*z* 1525.5, which represent FA accumulation and protease-induced softening, respectively. In the future, we need to identify these molecular ions, use these peptides as new indices, and test real fish samples to define the freshness of fish.

## Supplementary Materials

The following are available online at https://www.mdpi.com/2304-8158/9/4/402/s1, Figure S1: Sample conditions and mass spectra. Figure S2: Mass spectrum of autolysis peptides of protease.
