# Supplementary figures and images for: Application of Matrix-Assisted Laser Desorption/Ionization Mass Spectrometry Imaging for Evaluating the Quality of Fish Fillets
Source: Foods. 2020 Apr 1;9(4):402. doi: 10.3390/foods9040402 (PMC7230717; doi:10.3390/foods9040402)

# Protease derived peak

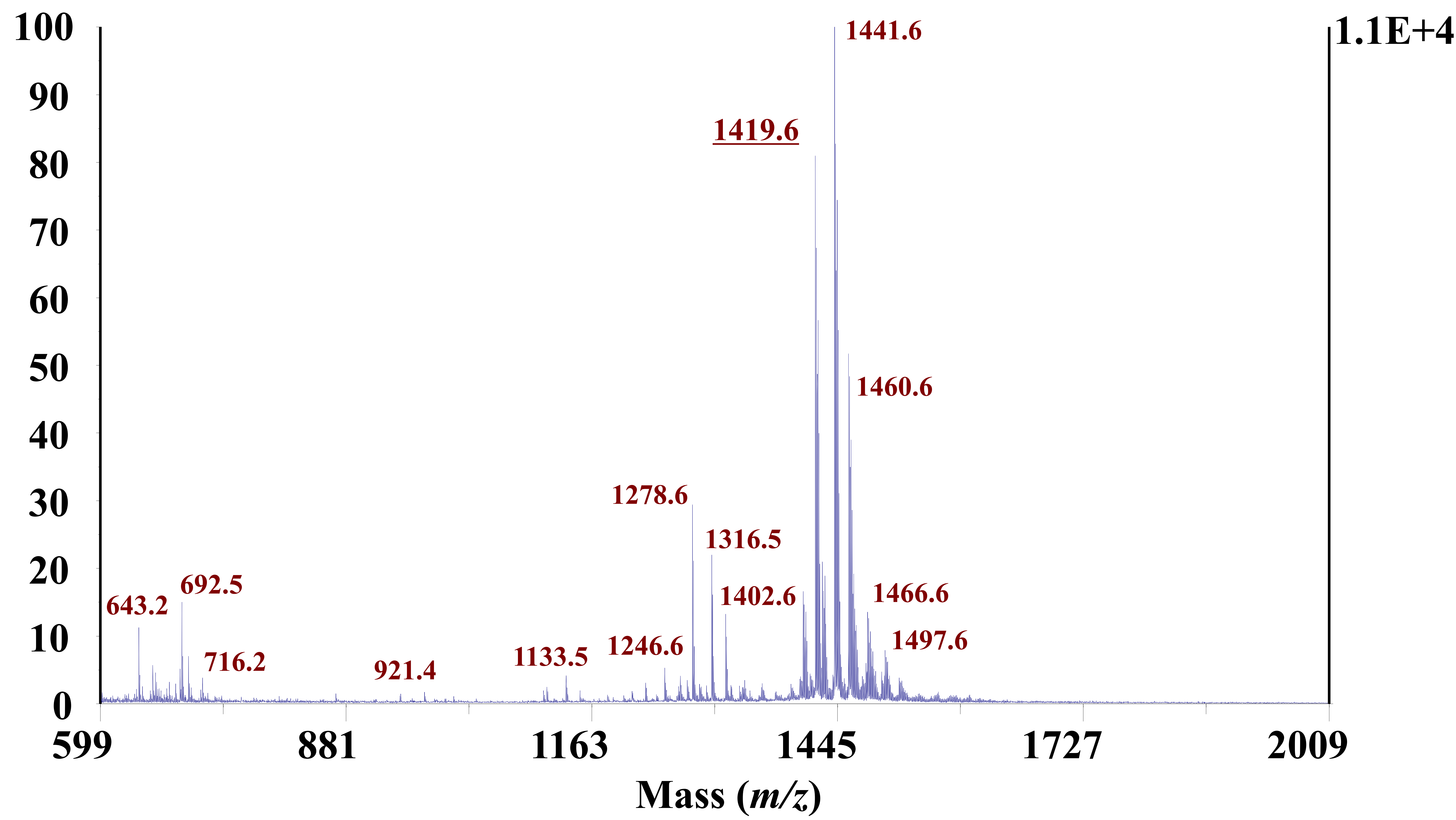

Supplemental figure2. Mass spectrum of autolysis peptides of protease.

Supplement: Supplementary file 1 [file foods-09-00402-s001.pdf]
